# Multimodal evaluation and etiology-oriented management of chronic intracranial venous hypertension

**DOI:** 10.3389/fneur.2026.1919940

**Published:** 2026-09-30

**Authors:** Jie Shen, Huabin Wang, Long Wang, Yifan Zhang, Sunhong Yan, Jia Wang

**Affiliations:** 1 Department of Neurology, The Second Affiliated Hospital of Anhui Medical University, Hefei, Anhui, China; 2 Anhui Provincial International Joint Research Center for Advanced Technology in Medical Imaging, School of Computer Science and Technology, Anhui University, Hefei, Anhui, China; 3 The Second Clinical Medical College, Anhui Medical University, Hefei, Anhui, China; 4 Department of Nephropathy, The Third Affiliated Hospital of Anhui Medical University, Hefei, Anhui Province, China

**Keywords:** chronic intracranial venous hypertension, high-resolution MRI, multimodal imaging, venous manometry, venous sinus stenosis, venous sinus stenting

## Abstract

Chronic intracranial venous hypertension (CIVHS) comprises a heterogeneous group of disorders characterized by impaired cerebral venous outflow and symptoms related to elevated intracranial pressure. Although venous sinus stenting is increasingly used for venous sinus stenosis with elevated trans-stenotic pressure gradients, venous sinus stenosis is an imaging phenotype rather than a single diagnosis. We retrospectively reviewed 13 consecutive patients with CIVHS who underwent clinical, imaging, and hemodynamic evaluation, including conventional neuroimaging, MR or CT venography, digital subtraction angiography, venous manometry when indicated, and selective high-resolution MRI. All patients initially presented to ophthalmology and had papilledema. Patients were classified according to presumed mechanisms of venous outflow impairment, and treatment was tailored to the anatomical substrate, hemodynamic findings, and clinical context. Six etiological categories were identified: intrinsic transverse sinus stenosis, extrinsic transverse-sigmoid sinus compression, shunt dependency syndrome, jugular foramen stenosis related to chronic organized thrombus, JAK2 V617F mutation-positive essential thrombocythemia with suspected microvascular venous congestion, and dialysis catheter-related central venous stenosis. An additional chronic cerebral venous sinus thrombosis case was included as an illustrative secondary comparator but was not classified as idiopathic intracranial hypertension. Treatments included venous sinus stenting, shunt-related management, hematologic-directed therapy, anticoagulation, acetazolamide, weight control, and conservative management. Among the 13 cohort patients with available follow-up, symptoms improved or remained clinically stable; Case 14 was lost to follow-up. No stent failure was documented during short-term follow-up, but long-term surveillance data were unavailable. These findings support an etiology-oriented, multimodal approach to chronic intracranial venous hypertension.

## 1 Introduction

Chronic intracranial venous hypertension (CIVHS) is an increasingly recognized clinical syndrome characterized by impaired cerebral venous outflow and symptoms related to elevated intracranial pressure (1, 2). Patients may present with headache, visual disturbance, papilledema, pulsatile tinnitus, or other nonspecific neurological symptoms (1, 2). In clinical practice, the condition is often evaluated in the context of idiopathic intracranial hypertension or venous sinus stenosis; however, these terms do not fully capture the diversity of underlying mechanisms (3, 4).

Venous sinus stenosis is frequently detected on MR venography, CT venography, or catheter venography (3, 5). When accompanied by appropriate clinical features and a trans-stenotic pressure gradient, venous sinus stenting may provide symptomatic relief in selected patients (6, 7). Nevertheless, venous sinus stenosis is not a single disease entity (3, 4). Similar imaging appearances may result from intrinsic sinus lesions, extrinsic compression, shunt-related venous remodeling, extracranial venous obstruction, systemic hematologic disease, or central venous stenosis (3–5, 8). Treating all such cases as equivalent may result in inappropriate stent placement or missed opportunities for more specific therapy (3–5).

Multimodal evaluation is therefore important for defining the mechanism of venous hypertension. Non-invasive venous imaging can identify the level and pattern of outflow impairment, catheter venography and venous manometry provide anatomical and hemodynamic information, and high-resolution MRI may offer additional lesion-level characterization in selected patients with intracranial venous sinus stenosis (8–10). In this setting, high-resolution MRI should be regarded as a targeted adjunct rather than a universal screening test for all patients with CIVHS.

This case series evaluates an etiology-oriented diagnostic and treatment strategy for CIVHS, emphasizing multimodal assessment and selective use of high-resolution MRI for intracranial venous sinus stenosis.

## 2 Materials and methods

### 2.1 Study design and patients

We performed a retrospective case series of 13 consecutive patients diagnosed with CIVHS who underwent multimodal venous evaluation at The Second Affiliated Hospital of Anhui Medical University between January 2021 and December 2025. All patients initially presented to ophthalmology and had papilledema documented at presentation. Inclusion criteria followed the Modified Dandy Criteria for idiopathic intracranial hypertension, with confirmed elevated opening pressure (>25 cmH2O) and compatible clinical or radiological findings. Patients were included if clinical, imaging, and treatment data were sufficient to support etiological classification and management review. Demographic data, clinical manifestations, non-invasive imaging findings, catheter venography findings, manometric measurements when available, high-resolution MRI findings when performed, treatment strategies, and follow-up outcomes were collected from medical records. A separate chronic cerebral venous sinus thrombosis case is presented as an illustrative secondary comparator because the thrombotic etiology was established; this patient was not included in the 13-patient idiopathic/etiology-oriented cohort summary.

### 2.2 Diagnostic evaluation

All patients underwent clinical assessment and conventional neuroimaging. Venous assessment included MR venography or CT venography, digital subtraction angiography, and venous manometry when clinically indicated. High-resolution MRI was selectively performed in patients with suspected or confirmed intracranial venous sinus stenosis to characterize lesion morphology and identify potential anatomical substrates of stenosis. In patients without primary intracranial venous sinus stenosis, etiological classification relied on the combined clinical history, conventional imaging, catheter venography, manometry when available, systemic evaluation, and relevant extracranial or central venous findings.

### 2.3 Etiological classification and treatment strategy

Patients were categorized according to the presumed mechanism of CIVHS. Treatment was individualized based on underlying etiology, lesion morphology, hemodynamic significance, symptoms, ophthalmological risk, response to medical treatment, and clinical context. Venous sinus pressures were measured by catheter manometry proximal and distal to the stenotic segment, and the trans-stenotic gradient was calculated as the pressure difference. In this retrospective cohort, venous sinus stenting using self-expanding stents (Precise, Cordis) was considered for patients with fixed stenosis and a gradient ≥8 mmHg. Alternative strategies, including shunt revision, medical therapy, anticoagulation, acetazolamide, treatment of systemic disease, or conservative management, were selected when the mechanism was not considered primarily stent responsive.

### 2.4 Outcome assessment

Clinical outcomes were assessed during follow-up using documentation of symptom change, visual status when available, postoperative intracranial pressure measurements when performed, and treatment-related complications. Symptomatic improvement was defined as patient-reported improvement in presenting symptoms after treatment.

## 3 Results

### 3.1 Patient characteristics

Thirteen patients with CIVHS were included. The median age was 37 years (interquartile range, 24–61 years), and 8 patients were female. Because the cohort was identified through ophthalmology presentation, all patients had papilledema. Other presenting manifestations included headache, decreased or blurred vision, transient visual obscurations, diplopia, floaters, and pulsatile tinnitus. Patient-level clinical characteristics, exact presenting visual symptoms, imaging-to-case mapping, treatment, and outcomes are provided in Supplementary Table S1. The additional illustrative chronic cerebral venous sinus thrombosis case (Case 1) was analyzed separately and was not included in these descriptive statistics.

### 3.2 Etiological categories

A chronic cerebral venous sinus thrombosis comparator, including treatment with venous sinus stenting, is illustrated in Figure 1. Within the 13-patient CIVHS cohort, six etiological categories were identified. Intrinsic transverse sinus stenosis caused by giant arachnoid granulations, fibrous septations, or brain herniation into arachnoid granulations is shown in Figure 2. JAK2 V617F mutation-positive essential thrombocythemia with suspected microvascular venous congestion and jugular foramen stenosis related to chronic organized thrombus are shown in Figure 3. Dialysis catheter-related central venous stenosis and shunt dependency syndrome are shown in Figure 4. A stented representative case of extrinsic-type transverse-sigmoid sinus stenosis is shown in Figure 5. Additional intrinsic and extrinsic angiographic patterns are compared in Figure 6. Supplementary Table S1 provides patient-level characteristics, presenting symptoms, imaging-to-case mapping, treatment, and outcomes.

**Figure 1:**
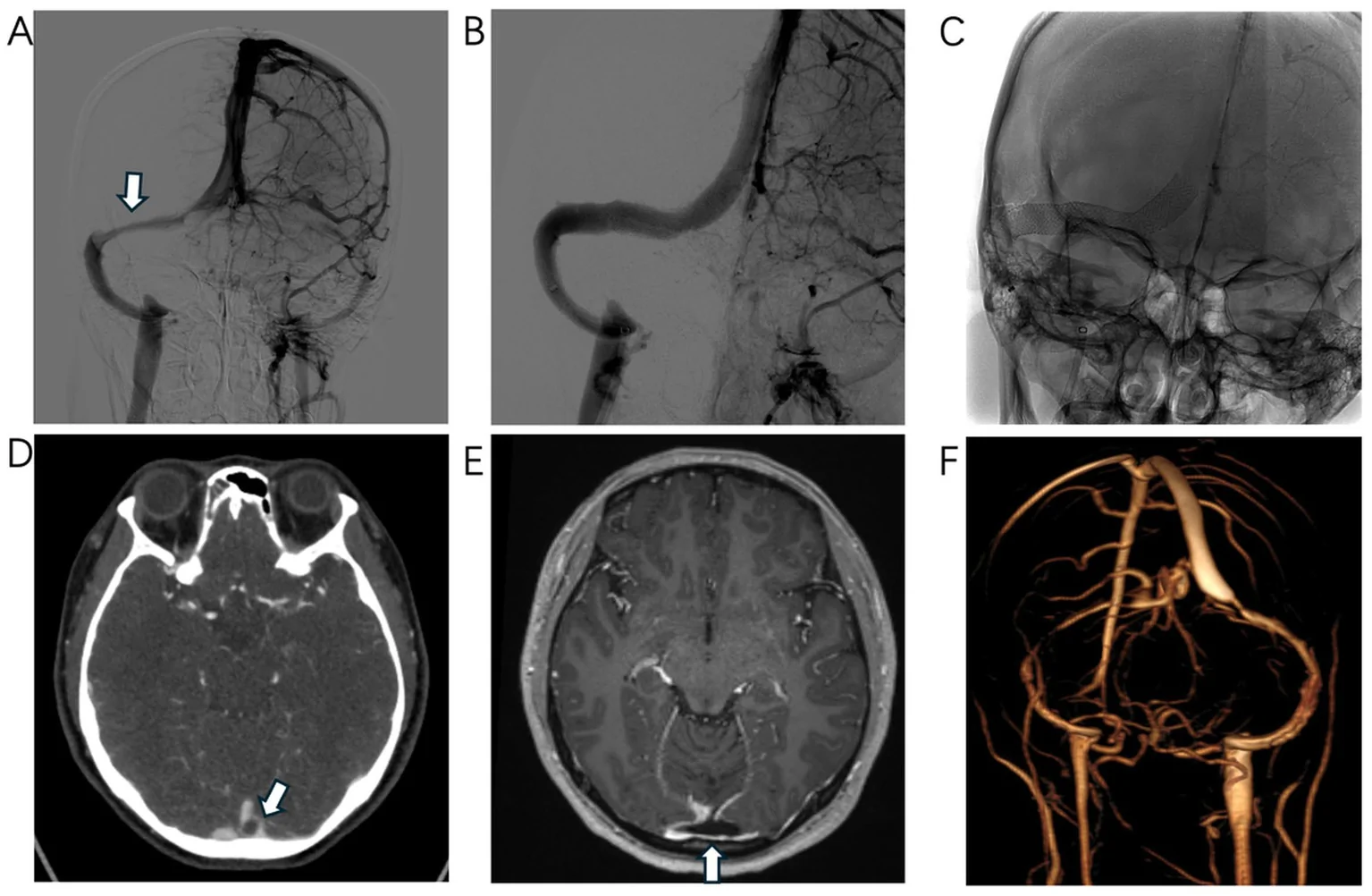
Venous sinus stenting for chronic cerebral venous sinus thrombosis with intracranial venous outflow obstruction (Case 1). Pre-stenting venous-phase digital subtraction angiography **(A)** demonstrates severe focal narrowing at the torcular region related to chronic thrombotic obstruction (arrow). Post-stenting venography **(B)** shows restoration of the venous sinus caliber and improved outflow, and the post-procedural angiographic image **(C)** demonstrates the deployed stent construct. Computed tomographic angiography **(D)** and contrast-enhanced magnetic resonance imaging **(E)** show the corresponding chronic thrombotic filling defect at the torcular Herophili (arrows). Pre-stenting three-dimensional magnetic resonance angiographic reconstruction **(F)** depicts the venous outflow obstruction and collateral venous drainage.

**Figure 2:**
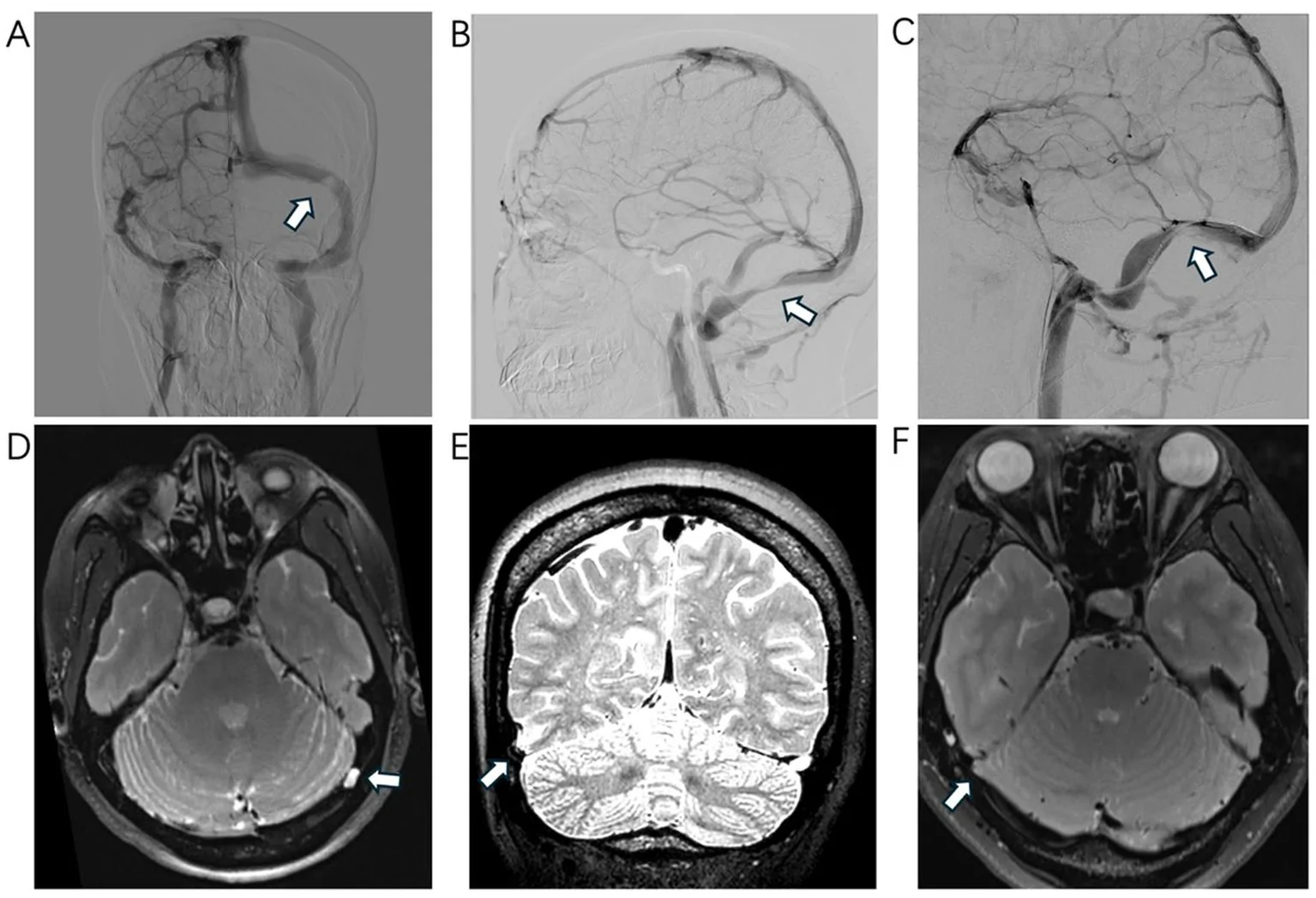
Representative imaging findings of intrinsic cerebral venous sinus stenosis. (**A,D**; Case 2) DSA demonstrates right transverse sinus hypoplasia and severe stenosis of the dominant left transverse sinus. HR-MRI identifies a giant arachnoid granulation as the cause of stenosis (arrow). (**B,E**; Case 3) DSA shows flow stratification at the stenotic segment of the right transverse sinus. HR-MRI demonstrates arachnoid granulations in the left transverse sinus and fibrous septations causing focal right transverse sinus stenosis (arrow). (**C,F**; Case 4) DSA shows focal transverse sinus stenosis. HR-MRI demonstrates cerebellar tissue protruding into the venous sinus lumen (arrow), consistent with brain herniation into an arachnoid granulation.

**Figure 3:**
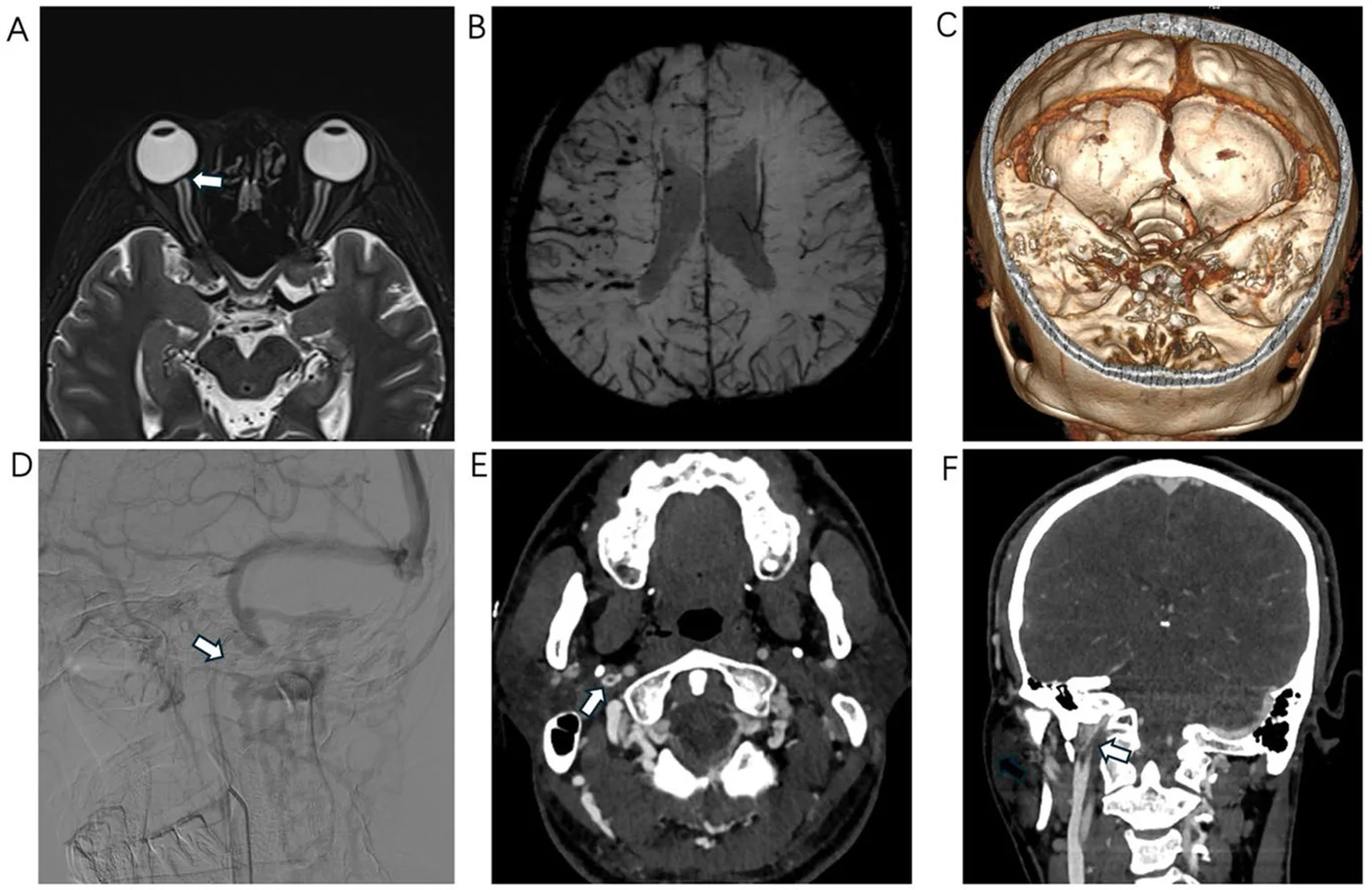
Imaging findings of JAK2 V617F mutation-positive essential thrombocythemia and jugular foramen stenosis with chronic internal jugular vein thrombosis. (**A–C**; Case 5) Axial T2-weighted MRI shows optic nerve sheath tortuosity and posterior globe flattening (**A**, arrow). Susceptibility-weighted imaging demonstrates multiple cerebral microbleeds in the right hemispheric white matter **(B)**. CTV shows patent intracranial dural venous sinuses **(C)**. (**D–F**; Case 6) DSA shows severe right internal jugular vein stenosis at the jugular foramen (**D**, arrow). CTV demonstrates a central filling defect consistent with chronic thrombosis (**E,F**, arrows), while the left internal jugular vein is not opacified.

**Figure 4:**
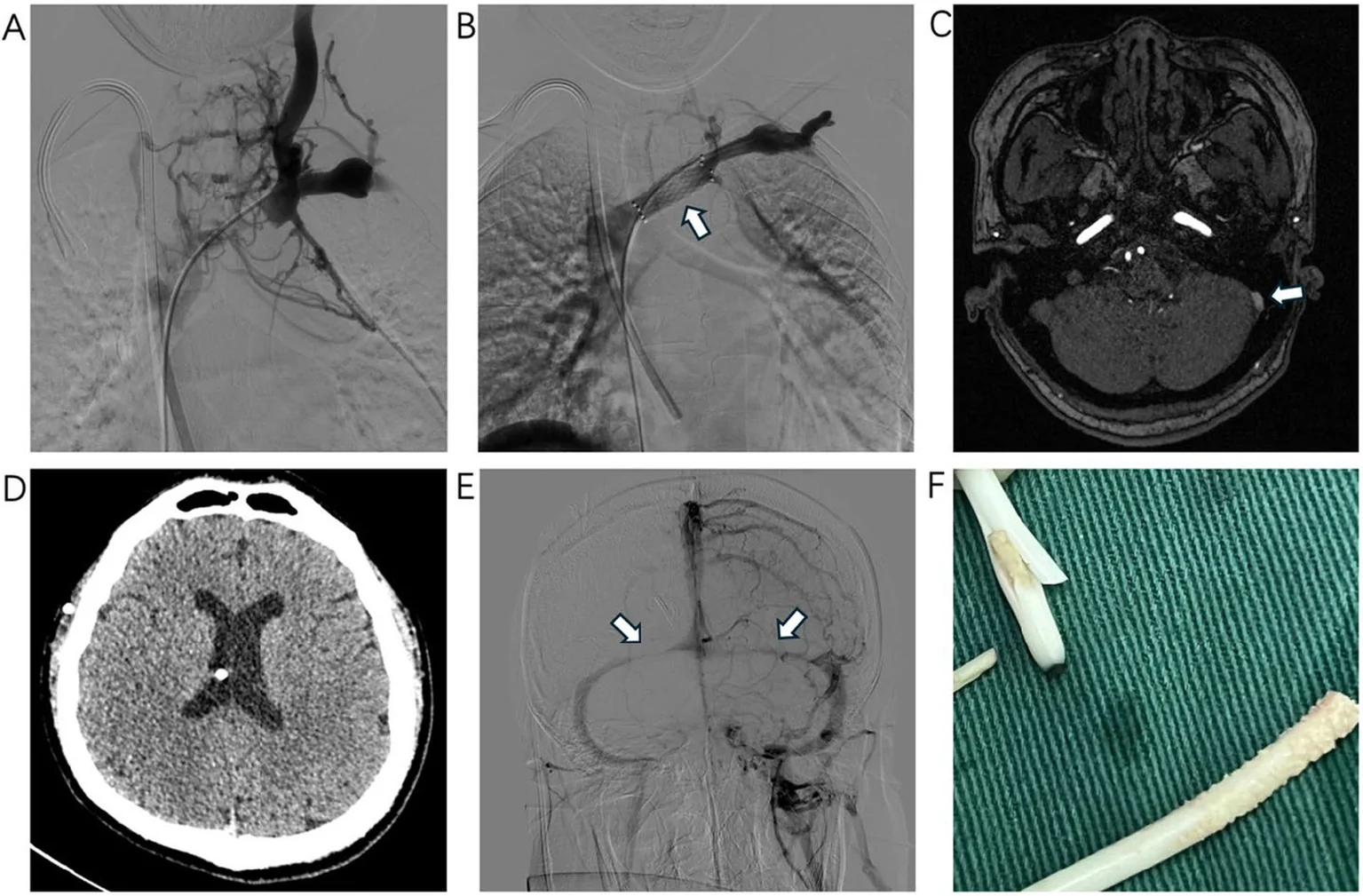
Imaging findings of central venous stenosis and shunt dependency syndrome. (**A–C**; Case 7) DSA demonstrates severe left brachiocephalic vein stenosis related to dialysis catheterization **(A)**. After stent placement, DSA shows successful recanalization and restoration of antegrade flow (**B**, arrow). TOF-MRA shows left sigmoid sinus hyperintensity caused by retrograde venous reflux (**C**, arrow). (**D–F**; Case 8) CT shows a ventriculoperitoneal shunt catheter and diffuse calvarial hyperostosis **(D)**. DSA demonstrates diffuse, smooth, multisegmental dural venous sinus stenosis (**E**, arrows). Intraoperative photography confirms obstruction of the distal peritoneal catheter tip **(F)**.

**Figure 5:**
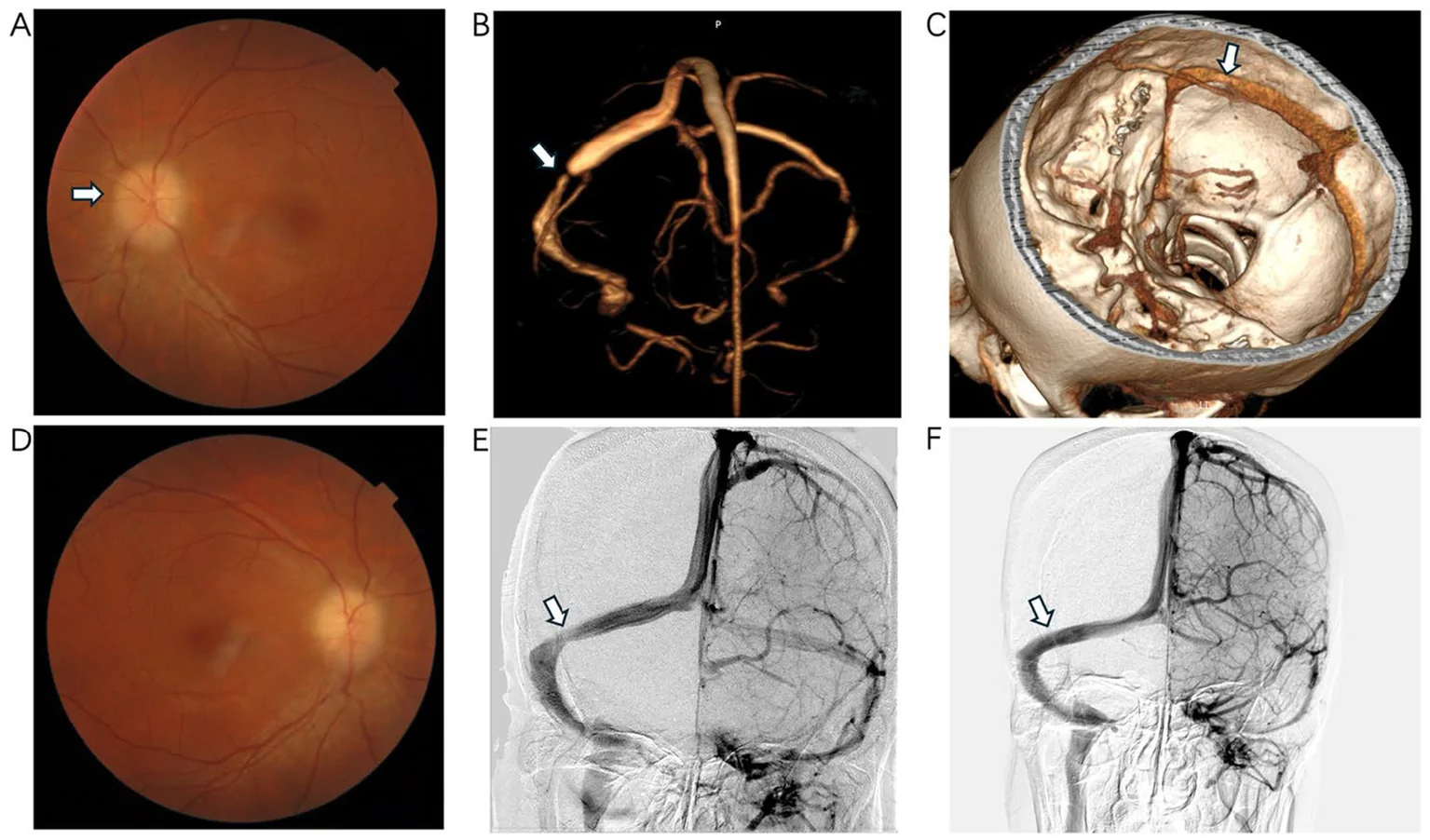
Multimodal findings and venous sinus stenting in a patient with extrinsic-type dural venous sinus stenosis (Case 9). Color fundus photographs of the left **(A)** and right **(D)** eyes demonstrate bilateral optic disc edema, with obscuration of the disc margins consistent with papilledema (arrow in **A**). Three-dimensional magnetic resonance angiographic **(B)** and computed tomographic venographic **(C)** reconstructions demonstrate smooth, tapered narrowing of the transverse-sigmoid sinus (arrows), consistent with extrinsic compression. Pre-stenting venous-phase digital subtraction angiography **(E)** confirms the focal stenosis (arrow). Post-stenting venography **(F)** shows restoration of the sinus caliber and unobstructed venous outflow across the treated segment (arrow).

**Figure 6:**
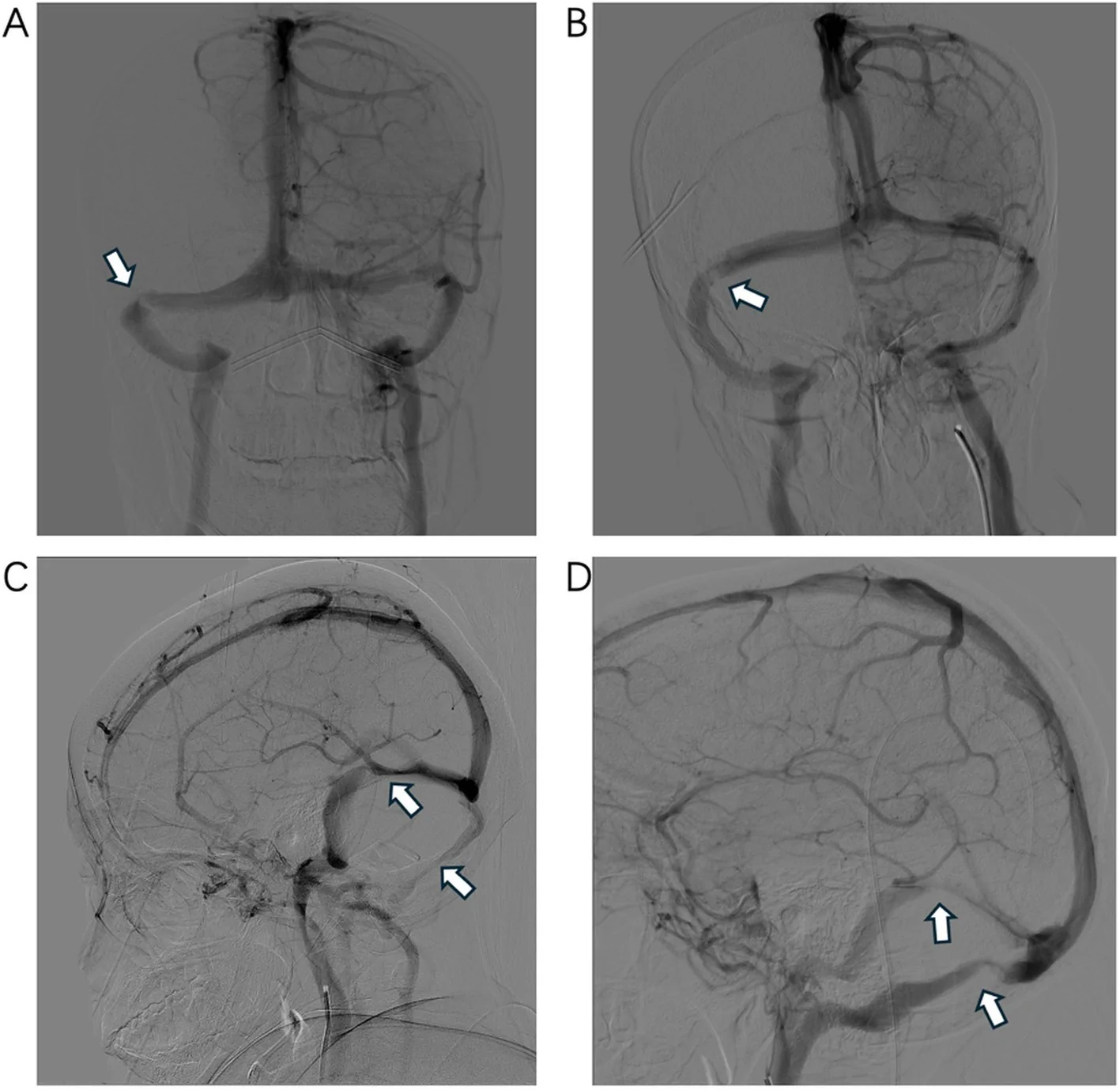
Angiographic patterns of intrinsic- and extrinsic-type dural venous sinus stenosis in four representative patients. Venous-phase digital subtraction angiography in two patients with intrinsic-type stenosis (**A**, Case 10; **B**, Case 11) demonstrates short, focal narrowing associated with discrete intraluminal filling defects (arrows). Venous-phase angiography in two patients with extrinsic-type stenosis (**C**, Case 12; **D**, Case 13) shows smooth, tapered segmental narrowing of the transverse-sigmoid sinuses without a discrete intraluminal filling defect (arrows).

### 3.3 Treatment and outcomes

Treatment was individualized according to the presumed mechanism of venous hypertension. Patients with fixed intracranial venous sinus stenosis and supportive clinical, anatomical, and hemodynamic findings underwent venous sinus stenting. Patients with shunt dependency syndrome received shunt-related management. The patient with jugular foramen stenosis related to chronic organized thrombus was managed with anticoagulation and acetazolamide. The patient with JAK2 V617F mutation-positive essential thrombocythemia received hematologic-directed therapy. For central venous stenosis related to prior dialysis catheterization, covered stent placement eliminated the pressure gradient and was associated with normalization of intracranial pressure. In the illustrative chronic cerebral venous sinus thrombosis case, stent placement restored the venous sinus lumen and antegrade drainage. Among 12 cohort patients with available follow-up, symptoms improved or remained stable; Case 14 was lost to follow-up. No stent failure or major treatment-related complication was documented during short-term follow-up. Long-term stent-patency and clinical follow-up data were unavailable.

## 4 Discussion

This case series highlights the etiological heterogeneity of CIVHS and supports the value of a multimodal, etiology-oriented diagnostic strategy. The key finding is that venous sinus stenosis should be regarded as an imaging phenotype rather than a uniform disease entity. Similar venographic appearances may reflect different mechanisms, each with distinct therapeutic implications (3–5).

Idiopathic intracranial hypertension is usually defined by clinical, neuro-ophthalmological, cerebrospinal fluid, and imaging criteria, but growing evidence suggests that venous outflow abnormalities, systemic factors, and patient phenotypes vary substantially across populations (1, 2, 11–14, 23). Epidemiological studies have demonstrated increasing disease burden and important associations with obesity, sleep apnea, and other systemic risk factors (11, 12, 15, 16). These observations support a shift from a purely syndrome-based framework toward mechanism-oriented evaluation in selected patients.

Venous sinus stenting has become an important treatment option for selected patients with intracranial venous hypertension and focal venous sinus stenosis. However, patient selection remains critical. The ≥8 mmHg trans-stenotic gradient used in this cohort reflects a commonly used historical selection threshold and a pragmatic concern that intervention across a very small gradient might provide less predictable postoperative benefit; it should not be interpreted as a prospectively validated biological cutoff (17). A prospective-registry analysis nevertheless reported comparable improvement in intracranial pressure, papilledema, and clinical symptoms after stenting among patients with gradients ≤4, 5–8, and >8 mmHg, suggesting that a low gradient alone should not automatically exclude an otherwise appropriate candidate (18). Gradient measurements can also vary with anesthesia, venous access, patient position, hydration, and technical factors. Accordingly, the gradient should be integrated with objective intracranial hypertension, visual risk, failure or intolerance of medical therapy, stenosis morphology, and the goal of restoring a normal-caliber lumen rather than used as an isolated binary rule. In the present series, only some patients had fixed intrinsic transverse sinus lesions that were considered directly stent-responsive. Others had shunt-related, extracranial, hematologic, or central venous causes that required alternative management.

Extrinsic-type dural venous sinus stenosis is an important counterpart to intrinsic stenosis. It typically appears as a smooth, tapered, often longer-segment narrowing caused by external compression of a collapsible dural sinus in the setting of elevated intracranial pressure, and it may participate in a positive-feedback loop in which elevated intracranial pressure narrows the sinus, raises upstream venous pressure, and further impairs cerebrospinal-fluid absorption (6, 8). One patient in the present cohort had extrinsic transverse-sigmoid sinus narrowing distal to the vein of Labbé entry points; CTV showed no corresponding arachnoid granulation or discrete intraluminal filling defect (Figure 5). Published data indicate that medically refractory patients with either intrinsic or extrinsic stenosis may benefit from restoration of the normal-caliber lumen by venous sinus stenting, although outcomes may differ between morphological subtypes (8, 19). Morphological classification should therefore inform, but not independently determine, treatment selection.

Cerebral venous sinus thrombosis is a well-established secondary cause of intracranial hypertension and is generally considered outside the idiopathic intracranial hypertension spectrum. It should be identified and managed as a defined thrombotic cerebrovascular disorder, with antithrombotic therapy as the treatment foundation (20). In selected chronic or organized thrombotic lesions with persistent focal venous outflow obstruction and intracranial hypertension, endovascular reconstruction may be considered after individualized multidisciplinary assessment. Figure 1 illustrates restoration of venous sinus patency after stent placement in a chronic thrombosis-related stenosis; this comparator was included to distinguish an etiologically defined secondary disorder from primary intrinsic or extrinsic stenosis.

The role of high-resolution MRI in this series was not as a universal screening tool for CIVHS, but as a targeted technique for patients with intracranial venous sinus stenosis. In these patients, high-resolution MRI added value by characterizing lesion morphology at the sinus level. It identified specific structural abnormalities such as giant arachnoid granulations, septation, organized intraluminal material, or brain herniation into arachnoid granulations (8–10, 21). This information may improve procedural planning and strengthen the rationale for stenting when the lesion is anatomically fixed and hemodynamically significant.

The concept of etiology-oriented treatment is particularly relevant because CIVHS may represent a final common pathway of several disorders. Shunt dependency syndrome, central venous stenosis from dialysis catheters, jugular foramen stenosis, and hematologic disease can all produce venous hypertension but are unlikely to respond uniformly to transverse sinus stenting. Recognizing these mechanisms is essential for selecting appropriate therapy.

For neurointerventional practice, these observations suggest that pressure gradients should not be interpreted in isolation. A lesion may be hemodynamically significant but still reflect a secondary or non-stent-responsive mechanism. Conversely, a fixed intrinsic stenotic lesion with supportive anatomical and hemodynamic features may represent an appropriate target for venous sinus stenting. Multimodal assessment allows these scenarios to be separated more reliably.

Papilledema remains a cardinal objective sign and often triggers further investigation. All patients in our cohort initially presented to ophthalmology and had papilledema, so this series does not include a subgroup with elevated pressure but no papilledema. Nevertheless, papilledema may be absent in a recognized minority of patients, and reliance on funduscopic findings alone can delay evaluation of patients with compatible symptoms and objectively elevated opening pressure (22). Patients without papilledema require confirmation of the pressure measurement, careful neuro-ophthalmological assessment, and exclusion of alternative causes; management should be guided by the complete clinical, imaging, and hemodynamic profile rather than by papilledema alone.

This study has limitations. The sample size was small, reflecting the rarity and heterogeneity of the condition, and the series was not intended to provide an exhaustive catalogue of CIVHS etiologies. Isolated congenital transverse sinus hypoplasia was not systematically evaluated or separately quantified in this retrospective cohort. Because all patients initially presented to ophthalmology with papilledema, the cohort may underrepresent patients with intracranial hypertension without papilledema. The chronic cerebral venous sinus thrombosis case was included only as an illustrative secondary comparator and was not part of the 13-patient cohort summary. The retrospective design may introduce selection bias, follow-up duration was variable, and Case 14 was lost to follow-up. No stent failure was documented during short-term follow-up, but surveillance imaging and formal long-term stent-patency assessment were unavailable. Not all patients underwent the same diagnostic tests, including high-resolution MRI, because imaging was selected according to the suspected mechanism and clinical need. Larger prospective cohorts with standardized imaging, manometry, neuro-ophthalmological outcomes, and long-term follow-up are required.

Despite these limitations, the study provides a practical framework for evaluating patients with CIVHS. An approach that integrates clinical history, conventional venous imaging, catheter venography, venous manometry, selective high-resolution MRI, and systemic evaluation may support more precise diagnosis, better treatment selection, and avoidance of unnecessary or inappropriate intervention.

## 5 Conclusion

Chronic intracranial venous hypertension is a heterogeneous syndrome with multiple potential etiologies. Multimodal assessment can distinguish intrinsic stenosis, extrinsic sinus compression, thrombotic obstruction, shunt-related remodeling, extracranial obstruction, and systemic contributors. Pressure gradients should be interpreted together with objective intracranial hypertension, visual risk, morphology, and response to medical treatment. Selective high-resolution MRI and etiology-oriented management may improve treatment selection and avoid inappropriate intervention.

## Data Availability

The data analyzed in this study are not publicly available because they contain potentially identifiable clinical information. De-identified data may be made available from the corresponding authors upon reasonable request and subject to institutional approval.

## Supplementary material

SUPPLEMENTARY Table S1 Summary of clinical characteristics, etiologies, imaging-to-case mapping, treatment, and outcomes.
