# Supplementary material for: Multimodal evaluation and etiology-oriented management of chronic intracranial venous hypertension
Source: Front Neurol. 2026 Sep 30;17:1919940. doi: 10.3389/fneur.2026.1919940 (PMC13661318; doi:10.3389/fneur.2026.1919940)
Supplement: Supplementary file 1 [file Table_1.docx]

**Supplementary Table S1**

*Summary of clinical characteristics, etiologies, imaging-to-case mapping, treatment, and outcomes*

| **Case** | **Age** | **BMI** | **Sex** | **Clinical presentation** | **Etiological category** | **Key diagnostic findings** | **Figure/MRI mapping** | **Etiology-oriented treatment** | **Clinical outcome** |
| --- | --- | --- | --- | --- | --- | --- | --- | --- | --- |
| 1* | 27 | 20.5 | F | Chronic headache, decreased vision, diplopia, and papilledema | Chronic cerebral venous sinus thrombosis | Chronic thrombotic filling defect at the torcular Herophili | Figure 1D-F; MRI in E; MRA in F | Venous sinus stenting | Marked reduction in intracranial pressure on postoperative lumbar puncture |
| 2 | 53 | 23.6 | F | Bilateral decreased vision with visual obscurations and papilledema | Intrinsic transverse sinus stenosis (giant arachnoid granulation) | Significant trans-stenotic gradient; HR-MRI supported intrinsic stenosis | Figure 2A,D; HR-MRI in D | Venous sinus stenting | Symptomatic improvement; intracranial pressure normalized on postoperative lumbar puncture |
| 3 | 43 | 22.6 | F | Left-eye visual obscuration, mildly decreased vision, and bilateral papilledema | Intrinsic transverse sinus stenosis (fibrous septations) | Significant trans-stenotic gradient; HR-MRI showed fibrous septations causing focal right transverse sinus stenosis | Figure 2B,E; HR-MRI in E | Venous sinus stenting | Symptomatic improvement; intracranial pressure normalized on postoperative lumbar puncture |
| 4 | 27 | 25.5 | F | Decreased vision, papilledema, and pulsatile tinnitus | Intrinsic transverse sinus stenosis (brain herniation into an arachnoid granulation) | Significant trans-stenotic gradient; HR-MRI showed brain parenchyma protruding into the right venous sinus lumen | Figure 2C,F; HR-MRI in F | Venous sinus stenting | Intracranial pressure normalized on postoperative lumbar puncture |
| 5 | 24 | 21.3 | M | Headache, blurred vision, floaters, and papilledema | JAK2 V617F mutation-positive essential thrombocythemia with microvascular pathology | Laboratory-confirmed essential thrombocythemia; susceptibility-weighted imaging showed multiple right-hemispheric white-matter microbleeds | Figure 3A-C; MRI/SWI in A-B | Medical and hematologic management | Symptomatic improvement |
| 6 | 37 | 24.5 | M | Headache, decreased vision, and papilledema | Jugular foramen stenosis | CTV showed a central filling defect in the right internal jugular vein; venography confirmed severe stenosis | Figure 3D-F; CTV in E-F | Anticoagulation and acetazolamide | Symptomatic improvement |
| 7 | 54 | 26.6 | F | Blurred vision, transient visual obscurations, and papilledema | Dialysis catheter-related central venous stenosis | Imaging and manometry identified central venous stenosis with a significant pressure gradient | Figure 4A-C; TOF-MRA in C | Covered stent placement | Intracranial pressure normalized |
| 8 | 29 | 30.1 | M | Headache, pulsatile tinnitus, and papilledema | Shunt dependency syndrome | History of ventriculoperitoneal shunting; venography showed diffuse, smooth, multisegmental dural venous sinus stenosis | Figure 4D-F; CT in D | Shunt-related management | Symptomatic improvement; intracranial pressure normalized on postoperative lumbar puncture |
| 9 | 39 | 23.4 | F | Headache, decreased vision, and papilledema | Extrinsic-type transverse-sigmoid sinus stenosis | Significant trans-stenotic gradient; MRV and CTV showed smooth, tapered extrinsic-type narrowing | Figure 5A-F; MRA/CTV in B-C | Venous sinus stenting | Symptomatic improvement; intracranial pressure normalized on postoperative lumbar puncture |
| 10 | 33 | 27.1 | M | Decreased vision and papilledema | Intrinsic transverse sinus stenosis (giant arachnoid granulation) | Multimodal evaluation showed hemodynamically relevant transverse sinus stenosis | Figure 6A | Venous sinus stenting | Intracranial pressure normalized on postoperative lumbar puncture |
| 11 | 61 | 24.4 | F | Bilateral blurred vision with dark shadows and papilledema | Intrinsic transverse sinus stenosis (giant arachnoid granulation) | Significant trans-stenotic gradient; HR-MRI supported intrinsic stenosis | Figure 6B | Patient declined surgery; acetazolamide | Stable without deterioration during outpatient follow-up |
| 12 | 36 | 28.7 | F | Decreased vision, transient visual obscurations, and papilledema | Extrinsic bilateral transverse-sigmoid sinus compression | Venous-phase angiography showed smooth, tapered segmental narrowing; HR-MRI supported extrinsic stenosis | Figure 6C | Weight control and acetazolamide | Stable without deterioration during outpatient follow-up |
| 13 | 28 | 32.0 | F | Intermittent blurred vision in the right eye and papilledema | Extrinsic bilateral transverse-sigmoid sinus compression | DSA showed smooth bilateral stenoses distal to the vein of Labbe; CTV showed no intraluminal filling defect | Figure 6D | Weight control and acetazolamide | Stable without deterioration during outpatient follow-up |
| 14 | 38 | 29.4 | M | Bilateral decreased vision and papilledema | Shunt dependency syndrome | History of arachnoid cystoperitoneal shunting; MRV showed diffuse, smooth, multisegmental dural venous sinus stenosis | Not illustrated | Shunt-related management | Lost to follow-up |

*Case 1 is an illustrative chronic cerebral venous sinus thrombosis comparator and is not included in the 13-patient cohort summary. All patients initially presented to ophthalmology and had papilledema. BMI, body mass index; CTV, computed tomographic venography; DSA, digital subtraction angiography; HR-MRI, high-resolution magnetic resonance imaging; MRA, magnetic resonance angiography; MRV, magnetic resonance venography; SWI, susceptibility-weighted imaging.
